# Two Consecutive Episodes of Severe Delayed Hemolytic Transfusion Reaction in a Sickle Cell Disease Patient

**DOI:** 10.1155/2020/2765012

**Published:** 2020-04-14

**Authors:** Clarisse Mpinganzima, Alf Haaland, Anne Guro Vreim Holm, Swee Lay Thein, Geir Erland Tjønnfjord, Per Ole Iversen

**Affiliations:** ^1^Department of Haematology, Oslo University Hospital, Oslo, Norway; ^2^Department of Orthopedic Surgery, Oslo University Hospital, Oslo, Norway; ^3^Sickle Cell Branch, National Heart Lung and Blood Institute, National Institutes of Health, Bethesda, MD, USA; ^4^Institute of Clinical Medicine, University of Oslo, Oslo, Norway; ^5^Department of Nutrition, Institute of Basic Medical Sciences, University of Oslo, Oslo, Norway; ^6^Division of Human Nutrition, Stellenbosch University, Tygerberg, South Africa

## Abstract

Patients with sickle cell disease (SCD) suffer from anemia and painful vaso-occlusive crisis (VOC) and sometimes need blood transfusions. Delayed hemolytic transfusion reaction (DHTR) is a rare life-threatening complication observed in SCD and mimics VOC. We describe a female SCD patient undergoing three surgical procedures during which DHTR developed following the first two. Prior to a planned tonsillectomy, she received transfusion and three days after surgery developed severe hemolysis as well as pain and respiratory symptoms. On suspicion of VOC, she received additional transfusions and became hemodynamically unstable, and her hemolytic anemia worsened. Gradually, she recovered and could be discharged after two weeks; DHTR was not suspected. Sixteen months later, an arthroplasty was performed due to avascular necrosis, and again she was transfused preoperatively. Similar to the initial surgery, she developed symptoms and signs of VOC after three days, but this time, DHTR was suspected and further transfusions were withheld. Although immunosuppressive medication did not alleviate the condition, she improved on combined treatment with darbepoietin, rituximab, and eculizumab. Six months later, a second arthroplasty was performed uneventfully after prophylaxis with rituximab and without transfusion. DHTR should be considered in the presence of severe, unexplained hemolysis following a recent transfusion, and additional transfusions in this setting should be given only on vital indication.

## 1. Introduction

Sickle cell disease (SCD) is an inherited hemoglobinopathy prevalent in equatorial regions such as sub-Saharan Africa. This is partly because carriers (heterozygotes) of the mutated sickle cell gene are protected against fatal complications of severe malaria [1]. The disease is characterized by hemolytic anemia, infections, and vaso-occlusive crisis (VOC) with acute as well as chronic pain, leading to significant lifelong morbidity and increased mortality.

In case of severe anemia, blood transfusions may be indicated in SCD. Repeated transfusions often result in the formation of anti-red blood cell (RBC) alloantibodies, and alloimmunization occurs more frequently in SCD patients than in other heavily transfused patient groups [2]. The prevalence of alloimmunization in SCD patients is reportedly between 30% and 50% [2, 3]. Delayed hemolytic transfusion reaction (DHTR) is a life-threatening complication due to alloimmunization. Generally, DHTR is rare, but it has been typically described among SCD patients, possibly due to mismatch between donor-RBC antigens (mainly Caucasian) and SCD-recipients (mainly Africans) [4]. There is to date no consensus definition of DHTR, but it is characterized by unequivocal evidence of severe hemolysis leading to a marked drop in hemoglobin levels below pre-fusion level, with or without detectable alloantibodies, and often appearing within 24 hours to 3 weeks after transfusion [5]. The true prevalence of DHTR among SCD patients is difficult to assess since this condition is probably often undiagnosed, but a prevalence ranging from 4% to 8% of transfused adult SCD patients has been reported with those receiving acute transfusions being at highest risk compared to those receiving chronic transfusions [6, 7].

Our knowledge about DHTR stems from case reports and case series usually involving only one DHTR event per patient. We here present an unusual case of repeated DHTRs without documented alloimmunization in a SCD patient treated at our unit on three different occasions where we suspect DHTR as the major underlying mechanism of the severe complications observed during the first two admissions. Consequently, a different approach to the third admission led to an uneventful clinical course.

## 2. Case Presentation

A 28-year-old Nigerian female with homozygous SCD became a regular outpatient at our department in 2013 after an uneventful (without any blood transfusion) pregnancy and birth in the USA. As a child, she had received several transfusions, apparently with no complications. She had the following blood type profile: 0; D+; C-; E-; c+; e+; K-; S-; in addition to anti-C and anti-E antibodies. She had never used any medication except folic acid.

### 2.1. Episode 1

**Figure 1:**
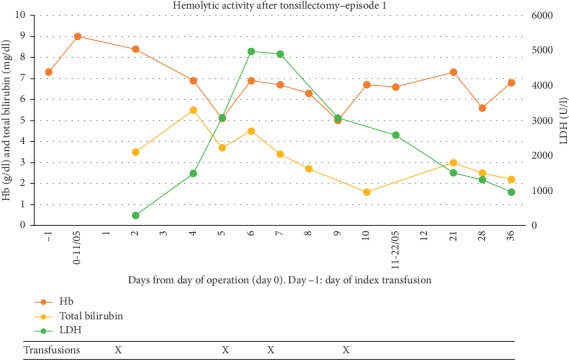
Time course of blood values obtained during and after the first episode. Reference ranges: Hb (11.7–15.3  g/dl); bilirubin (0.3–1.5  mg/dl); LDH (105–205 U/l).

In May 2017, she underwent an elective tonsillectomy at our hospital (a tertiary reference center in Norway; day 0). Except for routine pre-transfusion screening, neither extended screening for new alloantibodies nor a direct antiglobulin test was performed. On day −1, she received a scheduled transfusion of two cross-matched units of RBC with hemoglobin (Hb) increasing from a baseline value of 7 to 9.0 g/dl. After an uncomplicated procedure, she was transferred to a local hospital with an uneventful clinical course until day +4 when she developed generalized skeletal pain and fever, but no chest or respiratory symptoms. As Figure 1 shows, her Hb declined till day +5. There were concurrent signs of increased hemolysis with total bilirubin increasing to 5.5  mg/dl and lactate dehydrogenase (LDH) more than tripled in the same time period. On suspicion of VOC, she was on day +4 treated with analgesics, fluids, low-molecular-weight heparin, and antibiotics according to our guidelines. Her condition deteriorated within the next 24 hours, and she became increasingly respiratory distressed. A CT scan showed bilateral pulmonary infiltrates. Acute chest syndrome was suspected, and she received partial exchange transfusion with two cross-matched units of RBC, resulting in a transient Hb rise. Two additional units of RBC were administered, and the patient was transferred to the intensive care unit on day +6. Hb was then 6.9 g/dl, which over the next 6 hours fell to 5.1 g/dl. LDH peaked at 4,974 U/l, whereas haptoglobin was not detectable. Retrospectively, we noted a relative reticulocytopenia with a maximum value of 120 × 10^9^/l. She became hemodynamically unstable, and vasopressor support and two more units of RBC were given before she was intubated. She was extubated the following day, and over the next few days, her condition stabilized; the hemolytic parameters were markedly reduced by day +9, and she was discharged on day +13 with subsequent visits as an outpatient. All bacterial cultures from this admission were negative. Notably, during this first episode, DHTR was never suspected.

### 2.2. Episode 2

**Figure 2:**
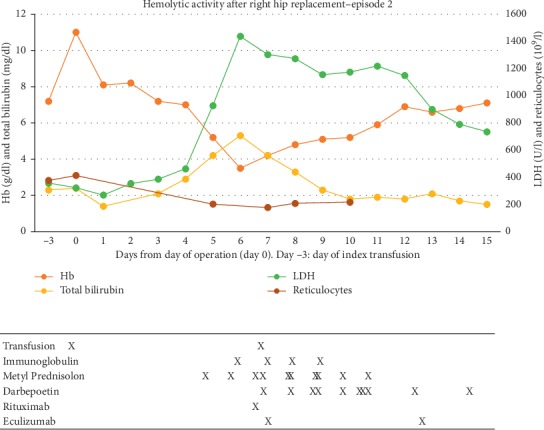
Time course of blood values obtained during and after the second episode. Reference range: reticulocytes (20–100 × 10^9^/l).

Due to avascular necrosis of her right hip, the patient underwent an arthroplasty in September 2018. On day −3 prior to surgery, she received a planned transfusion with three cross-matched units of RBC, raising her Hb to 11.0 g/dl. Her pre-transfusion screening revealed no new alloantibodies. After an uncomplicated orthopedic procedure on day 0, she was transferred to our department. On day +3, she developed skeletal pain but no symptoms related to the newly operated hip. On suspicion of VOC, treatment was initiated with fluids, analgesics, and antibiotics. As illustrated in Figure 2, her Hb fell gradually towards her baseline level without any obvious signs of bleeding or accelerated hemolysis during days 0 to +3. However, on day +5, she developed rapid and marked hemolysis with hemoglobinuria, Hb fell to 5.3  g/dl, and LDH rose to above 1,000 U/l. As noted in the first episode, there was a lack of adequate reticulocyte response with values falling from 413 to 177 × 10^9^/l. We suspected DHTR, and further transfusions were avoided. Intravenous immunoglobulin therapy (30 g daily) was initiated for 4  days. On day +6, Hb plummeted to 3.5  g/dl, and LDH and total bilirubin peaked at 1470 U/l and 5.3 mg/dl, respectively, and intravenous methylprednisolone (500  mg per day for 5 days) was administered. Screening for new alloantibodies and a direct antiglobulin test were both negative. Her HbA-fraction declined from 33.5% at day 0 to 12.3% on day +6. Using the nomogram proposed by Mekontso Dessap et al. [8], the likelihood of DHTR was considered as high. Because of the critically low Hb, she received one cross-matched unit of RBC on day +6. Rituximab (an anti-CD20 antibody, 1000  mg i.v.) was given together with the transfusion. Two single doses of eculizumab (an anti-complement factor 5 antibody, 900  mg i.v.) were administered on days +6 and +12, whereas the erythropoietin analogue darbepoietin (200  mg  s.c.) was given every second day from day +6 for a week. Over the next days, hemolysis declined, and her Hb stabilized. On day +4, complement factor C1q was 170 (reference 70–150) mg/l, increasing to 357 mg/l on day +6, whereas after the administration of eculizumab, markers of the classical, the lectin, and the alternative pathways for complement activation were all reduced towards undetectable levels. Despite signs of significant intravascular hemolysis, no serious organ dysfunction was recorded. She was discharged on day +15. On follow-up, she received pneumococcal and meningococcal vaccines. She had a full recovery and was thereafter not admitted for VOC, infections or transfusions.

### 2.3. Episode 3

**Figure 3:**
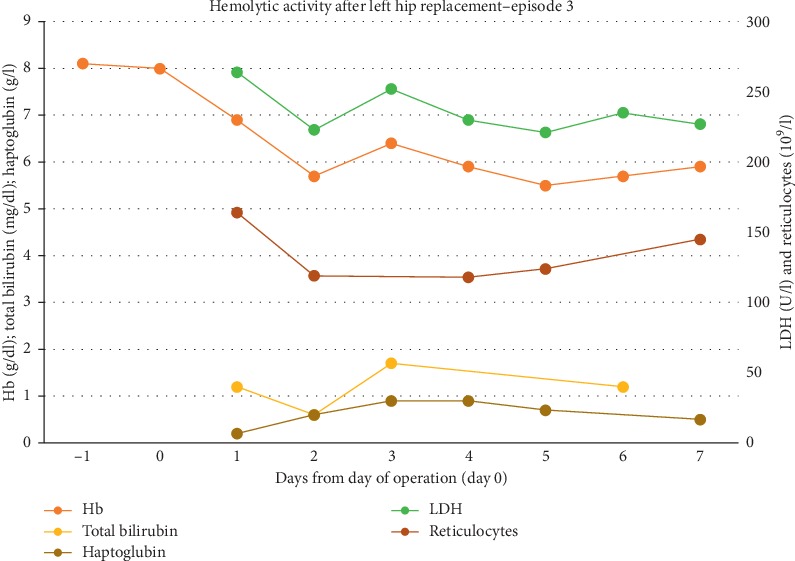
Time course of blood values obtained during and after the third episode. Reference range: haptoglobin (0.4–2.1 g/dl).

Due to progressive avascular necrosis of the contralateral hip, an arthroplasty was performed in March 2019. Screening for new alloantibodies and a direct antiglobulin test were both negative. As a preoperative strategy to optimize erythropoiesis, she received darbepoietin for two months as well as hydroxyurea, but no prophylactic transfusions. She maintained a preoperative Hb of about 8  g/dl, and she was also given one prophylactic dose of rituximab (1000  mg i.v.) 2.5 weeks before surgery. Both the surgery and the postoperative stay in hospital were uneventful and with no signs of hemolysis (Figure 3). She was discharged one week later. Regular follow-up visits at the outpatient clinic until December 2019 revealed no complications.

## 3. Discussion

We here present a SCD patient who received preoperative blood transfusions during two consecutive hospital admissions which were most likely complicated by DHTR. Although DHTR was not suspected during the first admission, we were alert to this complication during the second admission. Hence, we were able to provide her with adequate prophylactic treatment and avoided DHTR during her third admission.

Retrospectively, we believe the symptoms she presented during her postoperative hospital admission in 2017 represented the start of DHTR. A main challenge in identifying DHTR in SCD patients is that the symptoms often mimic VOC. In a large French observational study of about 3,000 SCD patients, the most common presenting symptoms and signs were hemoglobinuria (94%), pain (89%), fever (64%), and severe anemia (44%) [7]. Suppression of erythropoiesis reflected in reticulocytopenia (<150 × 10^9^/l) was observed in 40% of the patients. Progression to acute chest syndrome or even multi-organ failure was not uncommon in the most severe cases with an overall mortality of 6% [4]. Since transfusions are sometimes indicated in VOC, such treatment may further exacerbate hemolysis if DHTR is present [4–6].

Hyperhemolysis refers to the destruction of allogenic and autologous erythrocytes, potentially leading to severe and fatal hemolysis [6,7]. In line with this, our patient had a steep decline in Hb and spiking LDH on days +5 and +6 during the first and second episode, respectively. Alloantibody and direct antiglobulin screenings are imperative since it can aid in early identification of “missed” or evanescent antibodies. This is important also because patients may be treated at different institutions over the course of a lifetime, and without universal medical records, they may receive transfusions of blood with antibodies “lost to follow-up”. Moreover, follow-up screenings at regular intervals following a diagnosed DHTR will increase the likelihood of detecting new alloantibodies [9].

No new alloantibodies were detected in the acute setting during the second episode, and a direct antiglobulin test was negative. However, no repeated screenings were done between the first two episodes or after the second episode. Neither did we perform extended antibody screening for low-frequency antigens. Notably, hyperhemolysis may occur without newly formed alloantibodies and has been described in up to 1/3 of SCD patients [5, 8]. The pathophysiology of antibody negative DHTR is not fully understood, but may involve macrophage activation and/or heme involvement leading to endothelial damage and subsequent activation of the alternative complement pathway [10, 11].

There is little evidence-based data for the therapeutic approach to patients with suspected DHTR, and current treatment modalities are mainly based on expert recommendations [12]. Immunoglobulins or high-dose steroids are the recommended first-line therapy. Our patient received both agents as her hemolysis spiked, however, Hb still plummeted to 3.4  g/dl. We therefore chose to give a single transfusion at this critical anemia. We gave rituximab prior to transfusion to prevent new alloantibodies [13]. Relative reticulocytopenia secondary to suppressed erythropoiesis is a feature of DHTR, and it was observed in our patient during both episodes 1 and 2. To stimulate endogenous blood production, we administered an erythropoietin analogue. Eculizumab has been used in the management of patients with severe DHTR and severe hemolysis [14]. Our patient had elevated C1q indicating increased complement activation and thus received eculizumab along with rituximab. We do not know which agent contributed most to the resolution of the hemolysis, so we can only speculate that eculizumab was the most effective drug since it is known to result in rapid reduction of hemolysis [15].

DHTR is a rare life-threatening complication to transfusions in SCD patients. Our patient illustrates the dilemma that DHTR can be misdiagnosed as VOC, a complication of SCD that is much more common than DHTR. Hence, a DHTR diagnosis should always be considered in the presence of an unexplained increase in hemolysis following a recent transfusion, and further transfusions should be avoided unless life-threatening anemia is present.
